# Supplementary material for: Flicker light stimulation induces thalamocortical hyperconnectivity with LGN and higher-order thalamic nuclei
Source: Imaging Neurosci (Camb). 2023 Nov 23;1:imag-1-00033. doi: 10.1162/imag_a_00033 (PMC12007554; doi:10.1162/imag_a_00033)
Supplement: Supplementary Material [file imag_a_00033-supp.pdf]

Supplementary Information

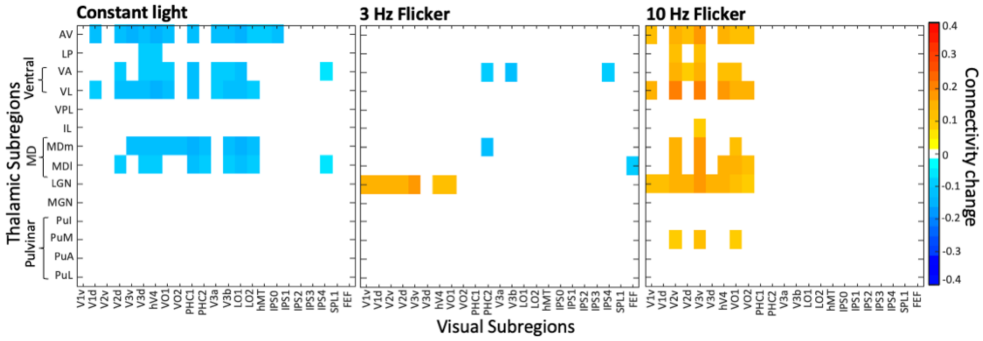

Figure S1. Connectivity matrices to show changes in thalamocortical connectivity without global signal regression (GSR) for all visual areas and thalamic nuclei during constant light, 3 Hz and 10 Hz FLS, calculated by subtracting the average of pre and post scans from connectivity matrices during the experimental conditions. A mask has been applied whereby only significant connectivity changes compared to baseline are shown, as determined by paired t-tests ($p < 0.01$). While the matrices appear dissimilar to data with GSR, they still show that 10 Hz FLS leads to stronger connectivity changes, especially for higher-order thalamic nuclei, while connectivity changes for LGN are comparable for 3 Hz and 10 Hz FLS. Moreover, the ventral stream visual areas display more increases in connectivity during 10 Hz than dorsal stream areas, which is also supported by findings with GSR. Lastly, the constant light condition induces thalamocortical hypoconnectivity, which is consistent with GSR results.

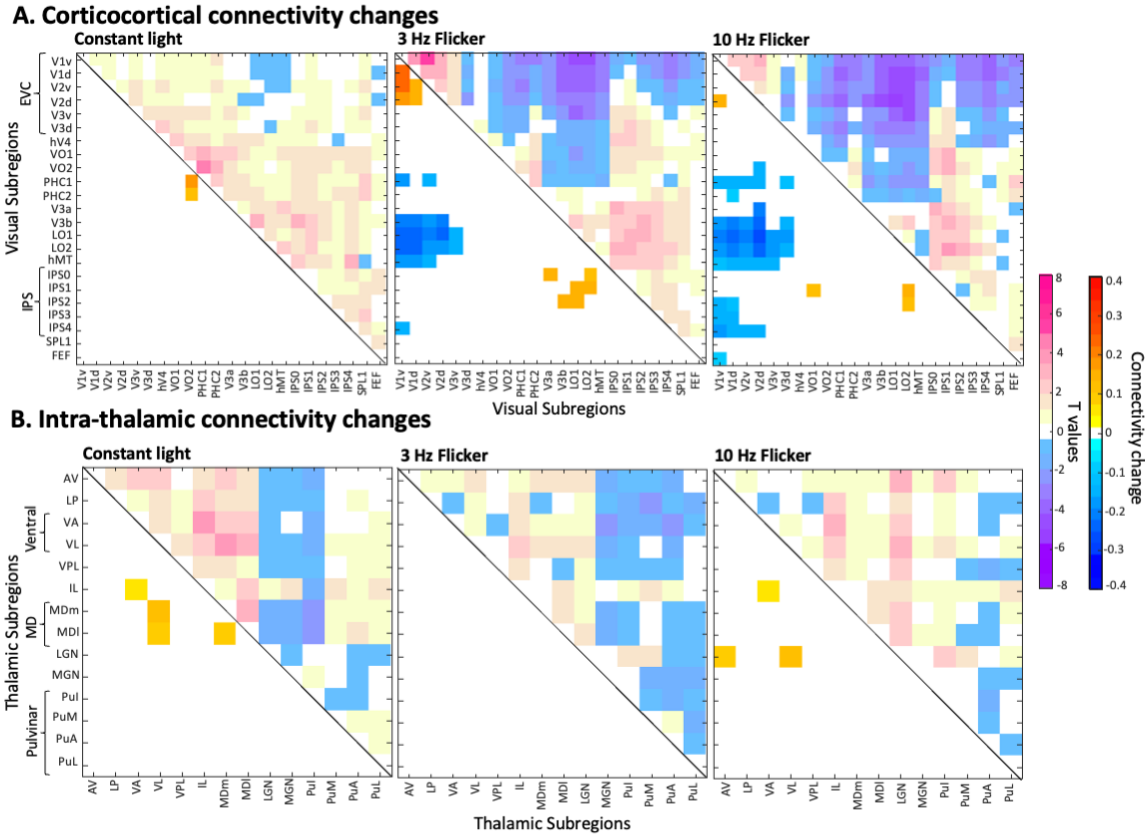

Figure S2. (A) Connectivity changes without GSR between visual areas during constant light, 3 Hz and 10 Hz FLS, as compared pre- and post- scans. Upper half of matrices show significance values of paired t-tests; Lower half show connectivity changes (masked at $p < 0.01$). (B) Connectivity changes without GSR within the thalamus. Hypoconnectivity between early visual areas and upstream visual areas are apparent for 3 Hz and 10 Hz FLS, which aligns with findings with GSR. There is little change in IPS connectivity, which otherwise appears strongly with GSR results.

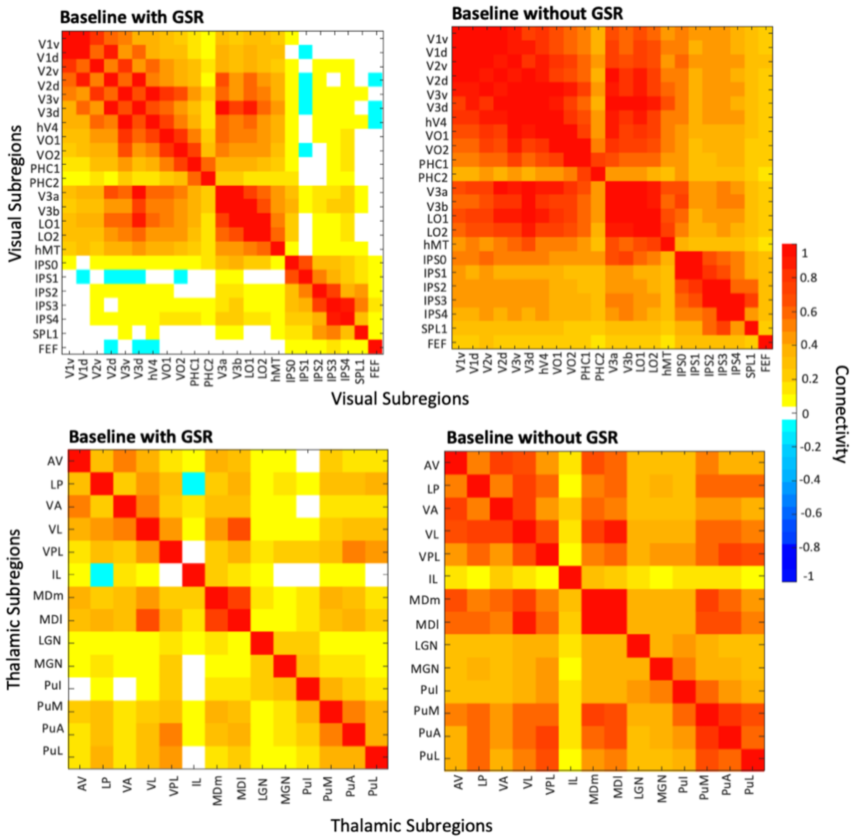

Figure S3. Intra-connectivity of (A) visual areas and (B) thalamic subregions with and without Global Signal Regression (GSR) during baseline, which constitutes the average of ROI-to-ROI correlational coefficients during pre and post resting-state closed-eye scans.

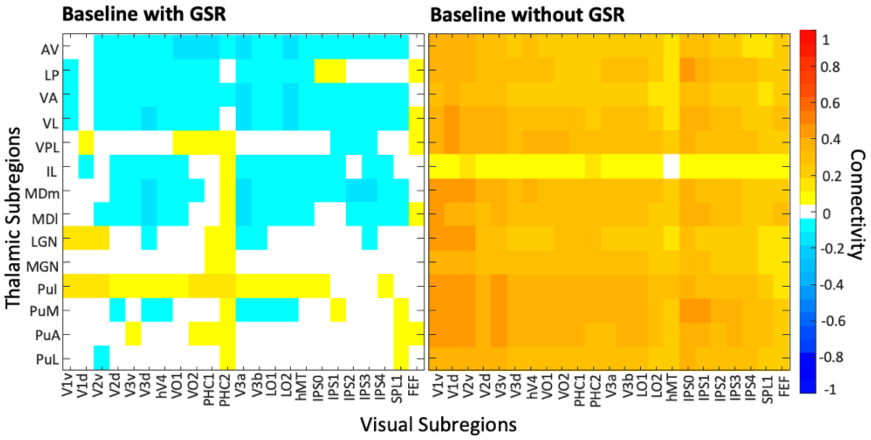

Figure S4. Thalamocortical connectivity during baseline (average of ROI-to-ROI correlational coefficients during pre and post resting-state closed-eye scans) calculated with and without GSR.

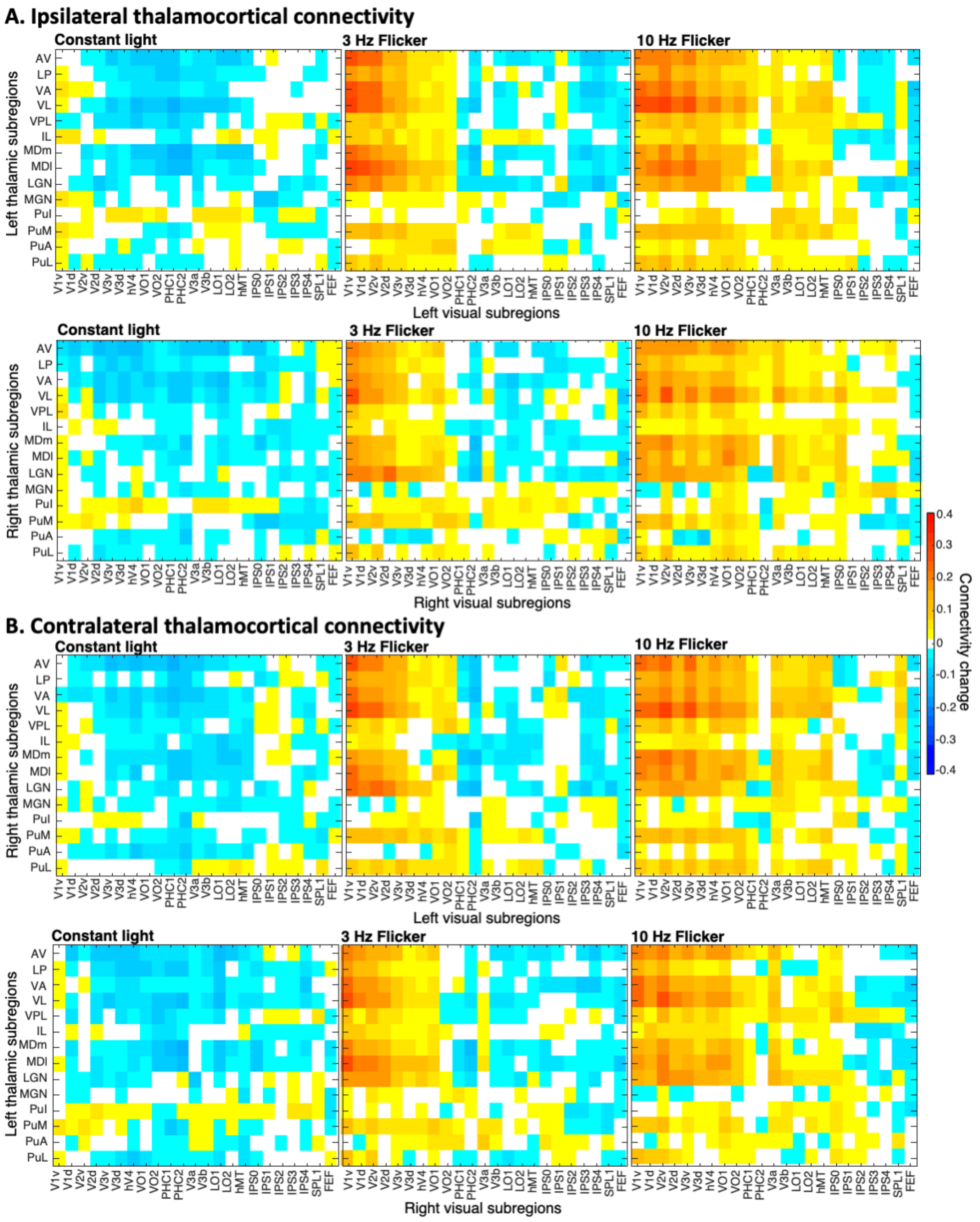

Figure S5. Connectivity matrices showing change in functional connectivity from baseline for constant light (left column), 3 Hz FLS (middle column) and 10 Hz FLS (right column) for all (A) ipsilateral thalamocortical connections between left hemispheric regions (upper row) and right hemispheric regions (lower row), and (B) contralateral connections between right thalamus and left cortex (upper row), and between left thalamus and right cortex (lower row). In sum, ipsilateral and contralateral thalamocortical connectivity patterns appear highly similar, which is expected as FLS was delivered equally to both eyes and thereby leads to a similar consequent pattern of connectivity in both hemispheres.

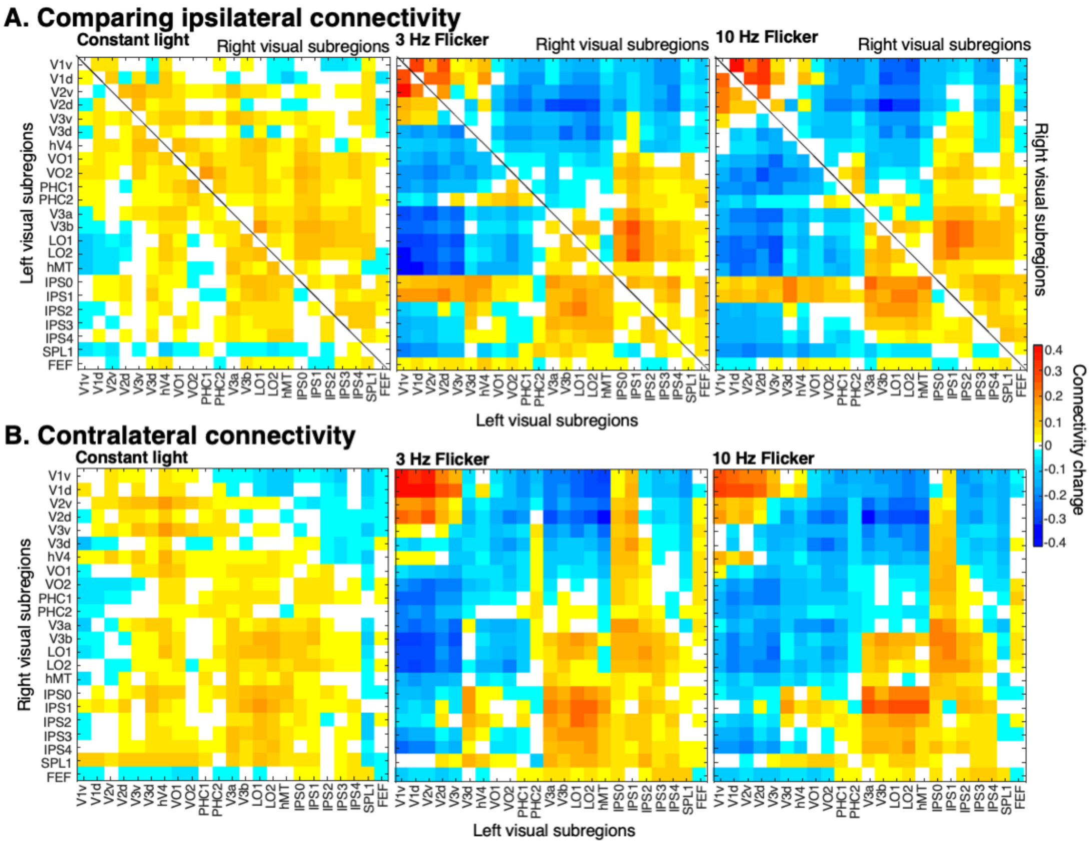

Figure S6. Connectivity matrices showing change in functional connectivity from baseline for constant light (left column), 3 Hz FLS (middle column) and 10 Hz FLS (right column) for all corticocortical connections within visual cortices for (A) left hemispheric regions (lower half) and right hemispheric regions (upper half), and (B) contralateral connections between left and right cortex. Ipsilateral and contralateral connectivity is highly similar across visual cortices, with the exception of left IPS0 and IPS1 which display notably stronger connectivity with all other ipsilateral and contralateral visual areas.

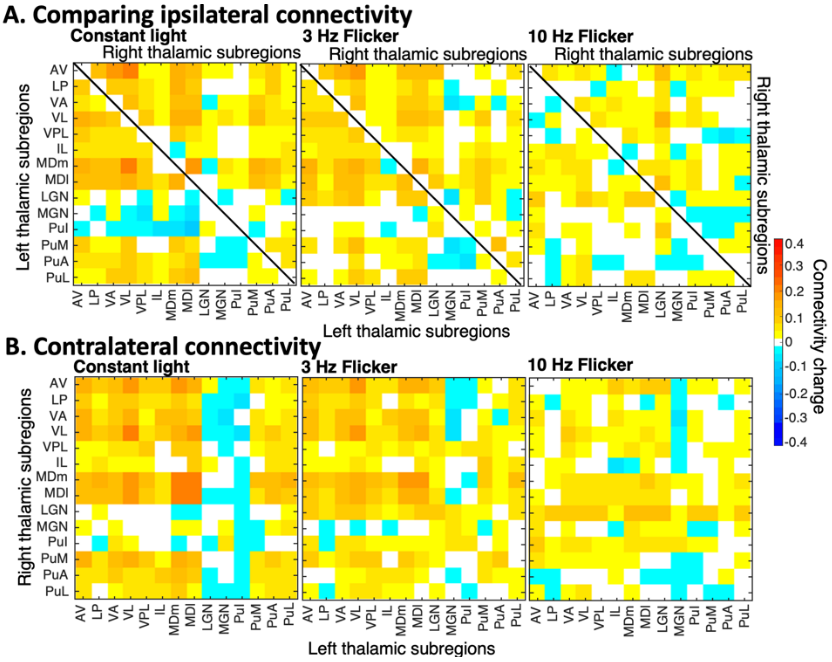

Figure S7. Connectivity matrices showing change in functional connectivity from baseline for constant light (left column), 3 Hz FLS (middle column) and 10 Hz FLS (right column) for all connections within (A) left thalamic regions (lower half) and right thalamic regions (upper half), and (B) contralateral connections between left and right thalamus.
